# Response of *Arabidopsis thaliana* to Flooding with Physical Flow

**DOI:** 10.3390/plants13243508

**Published:** 2024-12-16

**Authors:** Momoko Kaji, Kazuma Katano, Taufika Islam Anee, Hiroshi Nitta, Ryotaro Yamaji, Rio Shimizu, Shunsuke Shigaki, Hiroyuki Suzuki, Nobuhiro Suzuki

**Affiliations:** 1 National Institute of Technology, Ishikawa College, Tsubata 929-0392, Ishikawa, Japan; kj3030@ishikawa-nct.ac.jp (M.K.); hirofumi.nitta@fujita.co.jp (H.N.); yamaji1218@pref.ishikawa.lg.jp (R.Y.); 2 Division of Environmental Design, Graduate School of Science and Engineering, Kanazawa University, Kanazawa 920-1192, Ishikawa, Japan; 3 College of Life Sciences, Ritsumeikan University, Kusatsu 525-8577, Shiga, Japan; kzmktn-6@fc.ritsumei.ac.jp; 4 Department of Materials and Life Sciences, Faculty of Science and Technology, Sophia University, Chiyoda, Tokyo 102-8554, Japan; taufika_islam@sau.edu.bd; 5 Department of Agronomy, Faculty of Agriculture, Sher-e-Bangla Agricultural University, Dhaka 1207, Bangladesh; r-shimizu-3y2@eagle.sophia.ac.jp; 6 Principles of Informatics Research Division, National Institute of Informatics, Chiyoda, Tokyo 101-8430, Japan; shigaki@nii.ac.jp; 7 Department of Civil and Environmental Engineering, Faculty of Engineering, Hokkai-Gakuen University, Sapporo 062-8605, Hokkaido, Japan; hsuzuki@hgu.jp

**Keywords:** flooding, jasmonic acid (JA), pathogen defense, physical flow, respiratory burst oxidase homologue (RBOH), salicylic acid (SA), submergence

## Abstract

Flooding causes severe yield losses worldwide, making it urgent to enhance crop tolerance to this stress. Since natural flooding often involves physical flow, we hypothesized that the effects of submergence on plants could change when combined with physical flow. In this study, we analyzed the growth and transcriptome of *Arabidopsis thaliana* exposed to submergence or flooding with physical flow. Plants exposed to flooding with physical flow had smaller rosette diameters, especially at faster flow rates. Transcriptome analysis revealed that “defense response” transcripts were highly up-regulated in response to flooding with physical flow. In addition, up-regulation of transcripts encoding ROS-producing enzymes, SA synthesis, JA synthesis, and ethylene signaling was more pronounced under flooding with physical flow when compared to submergence. Although H_2_O_2_ accumulation changed in response to submergence or flooding with physical flow, it did not lead to lipid peroxidation, suggesting a role for ROS as signaling molecules under these conditions. Multiple regression analysis indicated possible links between rosette diameter under flooding with physical flow and the expression of *Rboh*s and SA synthesis transcripts. These findings suggest that pathogen defense responses, regulated by SA and ROS signaling, play crucial roles in plant responses to flooding with physical flow.

## 1. Introduction

Flooding can cause severe yield losses worldwide, resulting in devastating economic and sociological impacts [1]. Recent climate changes, such as rising sea levels and shifting rainfall patterns, can exacerbate the negative impact of flooding on crop yields [2,3]. Global warming increases air temperature, enabling the atmosphere to hold more moisture and leading to more intense rainfall [4]. Given these factors, the effects of flooding on plants and crops are likely to worsen due to global climate change. It is therefore urgent to establish strategies to enhance the tolerance of crops to flooding.

One of the most serious problems for plants caused by flooding is the disruption of gas diffusion between cells, leading to the inhibition of mitochondrial respiration and related metabolic processes [5]. Morphological and developmental alterations in plants under flooding are associated with their ability to adapt to low-oxygen conditions [1]. For example, plants can develop adventitious roots that acquire O_2_ and transport it to submerged roots [6,7]. Oxygen deficiency caused by flooding also enhances programmed cell death in the cortex, resulting in the production of air spaces, called aerenchyma, which are crucial for maintaining root respiration [7]. In addition, in rice, it has been demonstrated that internodal distances can elongate to prevent complete submergence of the plants [8].

Several key mechanisms governing plant responses to flooding have been elucidated in previous studies. In situations where respiration-dependent energy production is hampered, pyruvate fermentation becomes essential. Pyruvate Decarboxylase (PDC) and Lactate Dehydrogenase (LDH) convert pyruvate into acetaldehyde, subsequently reduced to ethanol by Alcoholic Dehydrogenase (ADH) to provide an alternative energy source [9,10,11,12]. Plant hormones also play crucial roles in the regulation of flood response of plants. Ethylene (ET) serves as a signal triggering acclimatory responses, including the regulation of transcripts encoding PDC and ADH [13]. Silencing Ethylene-Responsive Element-Binding Factor 2 (ERF2) resulted in the down-regulation of the expression of transcripts encoding PDC and ADH in Petunia, whereas the overexpression of ERF2 up-regulated the expression of these transcripts involved in pyruvate fermentation [14]. The significance of ET can also be extended to the formation of adventitious roots and aerenchyma [13,15,16]. It was demonstrated that expression of transcripts involved in ET production was up-regulated in flooded rice plants developing these structures [16]. In addition, ET promotes cell death necessary for aerenchyma formation [15]. These findings underscore the pivotal roles of ET signaling in flood responses in plants.

The roles of other plant hormones, such as gibberellin (GA), auxin (AU) and abscisic acid (ABA), in plant responses to flooding were also revealed in previous studies [17]. For example, exogenous applications of GA resulted in enhanced tolerance of plants to flooding accompanied by the attenuation of oxidative damage [18]. The production of ET under flooding facilitates AU transport, and accumulated AU, in turn, stimulates ET biosynthesis [19]. This reciprocal relationship promotes the transport of AU to the flooded parts of the plant, where cell division and adventitious root development occur [20]. In contrast, ABA was shown to negatively regulate formation of adventitious roots and aerenchyma via antagonistic manner with ET [1,21]. Furthermore, the level of salicylic acid (SA) and jasmonic acid (JA) increases in response to waterlogging. SA and JA can induce programmed cell death necessary for the formation of aerenchyma and production of ET [22,23,24]. These findings suggest that the response of plants to flooding is tightly regulated by the coordination of multiple plant hormone signals.

Flooding causes the inhibition of CO_2_ acquisition via the stomatal closure, negatively impacting mechanisms underlying photosynthesis [25]. Damage to photosynthesis as well as respiration increases cellular levels of reactive oxygen species (ROS), which can further harm cells under various abiotic stress conditions [25,26,27]. ROS scavenging enzymes, such as Superoxide Dismutase (SOD), Catalase (CAT) and Ascorbate Peroxidase (APX), can be activated in response to flooding to prevent excessive ROS accumulation [28], highlighting the importance of ROS scavenging systems for plant survival under flooding. However, transcripts encoding ROS-producing enzymes, Respiratory Burst Oxidase Homologues (RBOHs), were also shown to be up-regulated in response to flooding [29]. Despite their toxic potential, ROS play key roles in regulating numerous biological processes, involved in growth, development and responses to biotic and abiotic stresses [30]. Different RBOHs are expressed in various tissues and organs, with diverse roles evidenced in previous studies [31]. In *Arabidopsis thaliana*, RBOHD and RBOHF are key regulators of pathogen defense mechanisms [32,33,34]. Mutants deficient in these RBOHs showed inhibited ROS production and programmed cell death in response to pathogen attacks [32,33]. Further studies have demonstrated the roles of these RBOHs in regulating plant responses to abiotic stresses including waterlogging [35,36,37]. For example, a previous study demonstrated the significance of RBOH in rice in the regulation of ET-dependent aerenchyma formation [15]. In addition, ROS produced by RBOHD functions are implicated in mediating systemic responses to abiotic stresses including waterlogging as well as pathogen attack [38,39]. Although significant insights into the roles of RBOHs in regulating biotic and abiotic stress responses have been uncovered, their roles in regulating plant responses to flooding remain largely unknown. Superoxide anion (•O_2_⁻) generated by RBOH activity is converted to H_2_O_2_ through a reaction catalyzed by superoxide dismutase (SOD) [30]. H_2_O_2_ then acts as a signaling molecule, regulating a wide range of processes, including plant stress responses [40]. Due to its relative stability, H_2_O_2_ is a major ROS involved in signaling [40]. In contrast, excessive accumulation of ROS under stress conditions can lead to lipid peroxidation, resulting in the production of malondialdehyde (MDA) as a byproduct [41]. Thus, measuring H_2_O_2_ and MDA levels serves as an effective indicator of ROS signaling or oxidative damage in cells.

Despite advancements in understanding the mechanisms crucial for plant responses to flooding, there remains a significant gap between laboratory experiments and natural environments. In nature, flooding often coincides with other biotic or abiotic stresses, exacerbating its negative effects on plant growth [13]. The responses of plants to combined stresses cannot be inferred from responses to individual stresses, as discussed in a recent review [13]. In addition, natural flooding involves physical flow, which may elicit different plant responses compared to submergence alone. It has been demonstrated that physical stresses can impact the biological processes in plants. For example, statistical analysis and modeling indicated that tensile stress can alter the cell division plane orientation in *Arabidopsis thaliana* [42]. Furthermore, synthetic Arg-Gly-Asp (RGD) peptide, known for its role in cell adhesion in animal cells, dramatically inhibited the phosphorylation of MAPK-like cascades activated by shear stress and ROS production during shear stress in suspension-cultured *Taxus cuspidata* cells [43]. Despite the absence of a homologue of animal integrin, plant cells may use other RGD-binding proteins to recognize the RGD motif, which can be affected by shear stress. Based on these facts, we expected that the effects of submergence on plant can be altered when combined with physical flow. In this study, we analyzed growth parameters and the transcriptome of *Arabidopsis thaliana* exposed to submergence without physical flow and flooding with physical flow to characterize plant responses to flooding with physical flow. As no one has investigated the effects of flooding with physical flow on plants, we employed transcriptome analysis (RNA-Seq) to explore a broad range of processes that can be affected by flooding with physical flow.

## 2. Results

### 2.1. Effects of Flooding on Growth of Arabidopsis thaliana Plants

**Figure 1:**
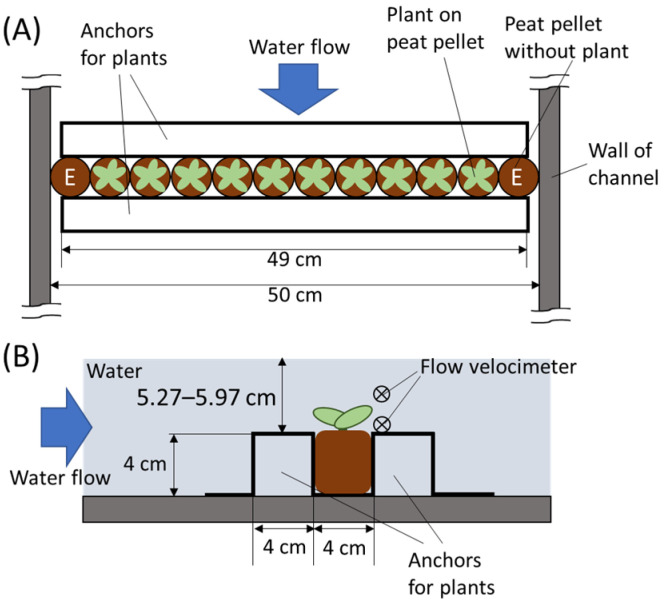
Schematic design of the channel to treat plants with flooding. (**A**) Top view, (**B**) side view.

**Figure 2:**
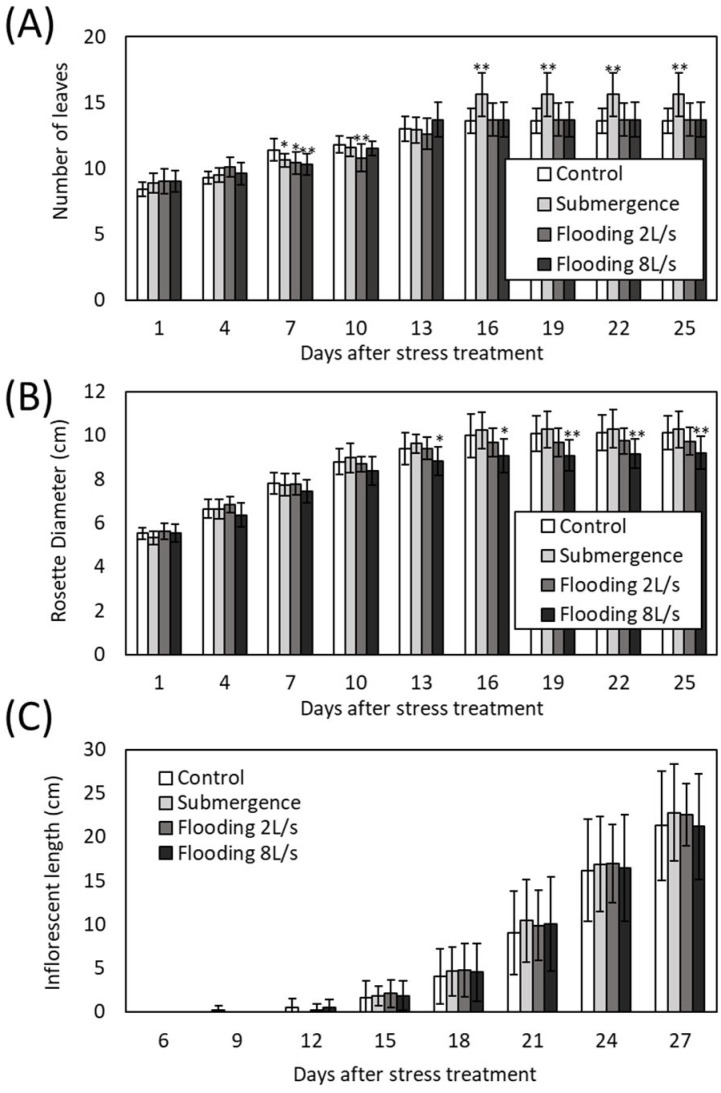
Growth characteristics of plants subjected to submergence or flooding with physical flow. (**A**) Number of leaves, (**B**) plant diameter and (**C**) inflorescent length. Error bars indicate standard deviation (*n* = 9–10). * and **: Student’s *t*-test significantly different at *p* < 0.05 and *p* < 0.01, respectively (compared to control).

In this study, we established the methods to apply flooding with physical flow to *Arabidopsis thaliana* plants by using a water channel (Figure 1 and Figure S1). Flooding was effectively implemented with flow rates of 2.0 and 8.0 L/s, maintaining a consistent water depth of approximately 10 cm (9.27–9.97 cm; Figure 1B). This suggests that the water pressure exerted on the plants was nearly uniform across all stress conditions. For comparison, the plants were also submerged into water at a depth of 10 cm in parallel in a plastic container. To assess the effects of flooding with physical flow on growth of *Arabidopsis thaliana* plants, we monitored number of leaves, rosette diameter and inflorescent length every 3 days for 25–27 days following the application of flooding with physical flow or submergence. The number of leaves decreased in plants exposed to stress at both 7 and 10 days post-treatment. However, from 16 days onwards, the plants exposed to submergence exhibited a significant increase in leaf number, whereas those subjected to flooding with physical flow did not (Figure 2A). In contrast, plants exposed to flooding with physical flow exhibited a smaller rosette diameter compared to those kept as controls or exposed to submergence. Notably, exposure to faster flow rate resulted in significantly smaller rosette diameter compared to controls from 13 days post-treatment (Figure 2B). However, neither flooding with physical flow nor submergence influenced the inflorescent length, nor did the timing of state transition from vegetative stage to reproductive stage (Figure 2C). These results suggest that *Arabidopsis thaliana* plants might differently respond to submergence and flooding with physical flow.

### 2.2. Transcriptome Analysis of Arabidopsis thaliana Plants Exposed to Flooding with Physical Flow

To explore the mechanisms through which plants respond to flooding with physical flow, we conducted a transcriptome analysis of 4-week-old plants subjected to either submergence or flooding with flow rate of 8 L/s. Our analysis yielded 41,763,964–70,107,698 input reads from the cDNA libraries (Table S1). Of these, 39,743,524–66,405,340 reads were mapped to the *Arabidopsis thaliana* reference genome (TAIR10). To provide an overview of this analysis, MA plots were generated (Figure S2). The fold change of up-regulated transcripts tended to be higher than that of down-regulated transcripts, regardless of whether plants were subjected to submergence or flooding.

**Figure 3:**
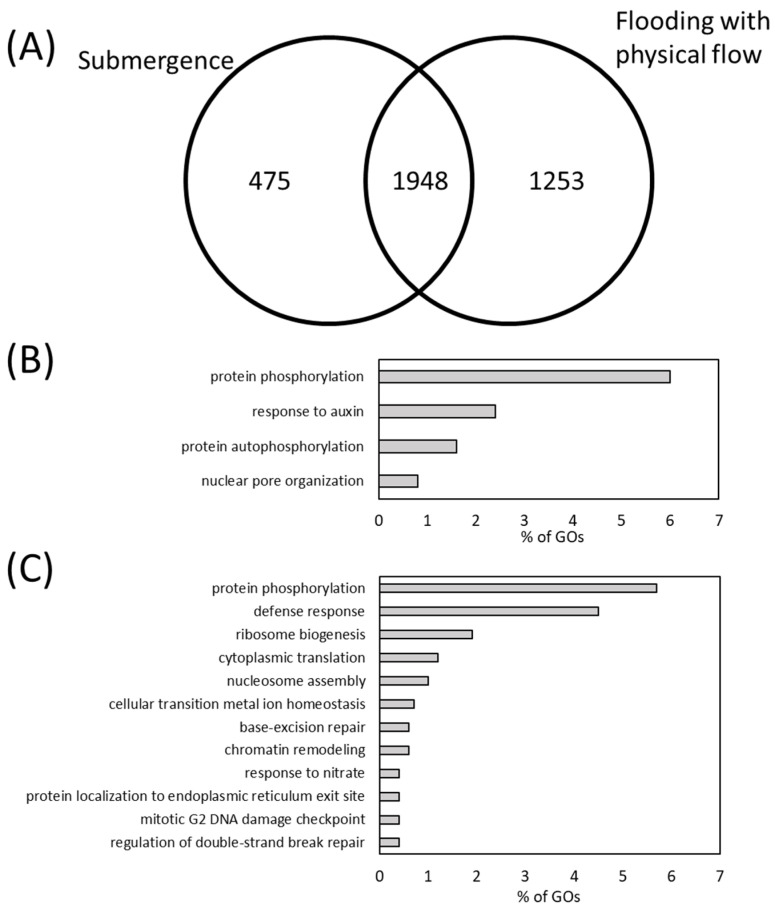
Characteristics of transcripts up-regulated in response to flooding with physical flow. (**A**) Venn diagram showing the overlap between transcripts up-regulated in response to submergence or flooding with physical flow. (**B**,**C**) Gene Ontology (GO) terms of “biological processes” represented in transcripts specifically up-regulated in response to submergence (**B**) or flooding with physical flow (**C**).

The results revealed that 2423 and 3201 transcripts were significantly up-regulated in response to submergence and flooding with physical flow, respectively. Among them, 475 transcripts were uniquely up-regulated during submergence, and 1253 transcripts were exclusive to flooding with physical flow (Figure 3A, Tables S2–S4). The transcripts up-regulated specifically in response to submergence predominantly fell into the Gene Ontology (GO) terms associated with biological process, such as “protein phosphorylation”, “response to auxin”, “protein autophosphorylation” and “nuclear pore organization” (Figure 3B, Tables S5 and S6). In addition, transcripts that are assigned to GO terms “plasma membrane”, “plant-type cell wall” and “nuclear pore inner ring” were also highly represented among those exclusively up-regulated during submergence (Tables S5 and S6). These results indicate the potential roles of protein phosphorylations and cell wall maintenance in the regulation of plant response to submergence. This speculation can also be supported by the high representation of transcripts that are assigned to GO terms, “kinase activity” and “pectin acetylesterase activity” (Tables S5 and S6). The GO terms highly represented among the transcripts specifically up-regulated in response to flooding with physical flow differed from those up-regulated in response to submergence (Figure 3B,C, Tables S7 and S8). Transcripts unique to flooding with physical flow fell into a wider array of GO terms associated with biological processes, including “defense response”, “ribosome biogenesis”, “regulation of double-strand break repair” and “chromatin remodeling” as well as “protein phosphorylation” (Figure 3C, Tables S7 and S8). In addition, GO terms associated with cellular component and molecular function underscore the roles of modulation of carbohydrate metabolisms, and support the importance of protein phosphorylation, ribosome biogenesis and defense responses (Tables S7 and S8). Furthermore, GO terms associated with pathogen responses and phosphorylation, including “defense response” and “protein phosphorylation”, were also highly represented in transcripts commonly up-regulated in response to submergence and flooding (Table S9).

The defense response transcripts specifically up-regulated in response to flooding with physical flow include many encoding R protein, such as Toll-Interleukin-1 Receptor/Nucleotide-Binding Site/Leucine-Rice-Repeat (TIR-NBS-LRR) and Coiled-Coil/Nucleotide-Binding Site/Leucine-Rice-Repeat CC-NBS-LRR class proteins (Tables S3 and S8). These proteins are well known for recognizing pathogen infection and activating downstream responses [44,45]. In addition, Jasmonate ZIM-domain protein (AT3G22275, also known as JAZ13) which is known to function as negative regulator of Jasmonic acid signaling [46], was also highly up-regulated specifically in response to flooding with physical flow. These facts suggest that strict modulation of pathogen defense might be required for the plant responses to flooding with physical flow.

In response to submergence and flooding, 1929 and 2755 transcripts were significantly down-regulated, respectively. Among them, 584 transcripts were uniquely down-regulated during submergence, and 1410 transcripts were exclusive to flooding (Figure S3A, Tables S10–S12). Transcripts that are assigned to GO terms associated with functions of the chloroplasts and mitochondria were highly represented in those solely down-regulated by submergence (Tables S13 and S14), suggesting the potential inhibition of photosynthesis and respiration during this stress. GO terms represented among the transcripts specifically down-regulated in response to flooding differ from those down-regulated by submergence (Figure S3B, Tables S15 and S16). These transcripts exclusively down-regulated by flooding fell into a wide array of GOs associated with processes such as protein ubiquitination, RNA processing, transcription and transport. In addition, GO terms related to post-translational modifications of proteins as well as ubiquitination and RNA processing were highly represented among the transcripts commonly down-regulated in response to submergence and flooding (Table S17).

The transcripts associated with ubiquitination that are specifically down-regulated in response to flooding with physical flow include ones encoding Phototropic-Responsive NPH3 family protein (AT1G50280), also known as BTB/POZ Protein Hypersensitive to ABA 1 (BPH1). This protein inhibits ABA signaling, and its deficiency in *Arabidopsis thaliana* resulted in enhanced drought tolerance [47]. The transcript encoding RING/U-box Superfamily Protein (AT1G04360), also known as Arabidopsis Toxicos En Levadura 1 (ATL1), was also down-regulated in response to flooding with physical flow. In a previous study, the overexpression of ATL1 resulted in induction of cell death as well as growth retardation [48]. The down-regulation of these transcripts suggests that mechanisms underlying responses to other stresses need to be modulated in the regulation of plant responses to flooding with physical flow.

Taken together, these results suggest that plants respond to flooding with physical flow via distinct mechanisms from that regulating submergence.

### 2.3. Involvement of Defense Mechanisms and Its Regulatory Plant Hormone in the Response of Arabidopsis thaliana to Flooding with Physical Flow

**Figure 4:**
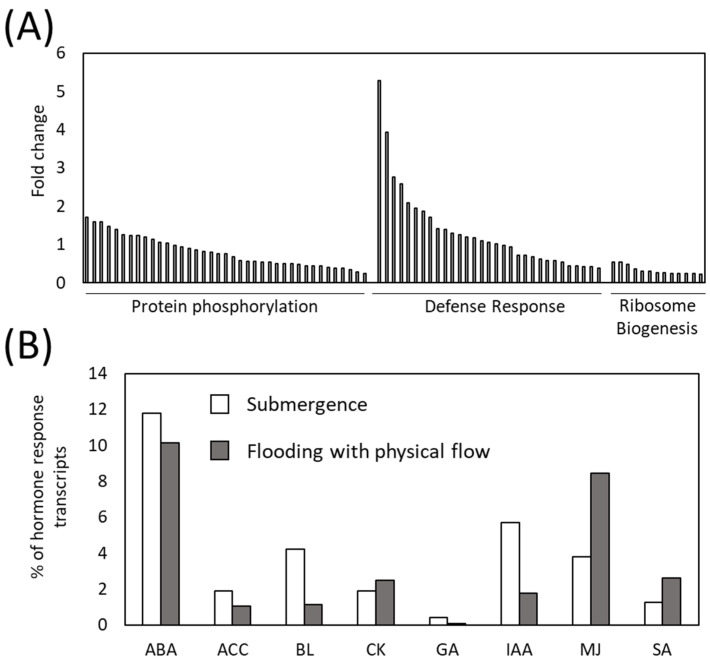
Involvement of pathogen defense pathways in response of plants to flooding with physical flow. (**A**) Fold change of transcripts belong to Gene Ontologies (GOs) that are highly represented in the transcripts specifically up-regulated in response to flooding with physical flow. (**B**) Proportion of hormone response transcripts among the transcripts specifically up-regulated in response to submergence or flooding with physical flow. ABA: abscisic acid, ACC: 1-aminocyclopropane-1-carboxylate, BL: brassinosteroids, CK: Cytokinin, GA: gibberellic acid, IAA: indole-3-acetic acid, MJ: methyl jasmonate, SA: salicylic acid.

We found that transcripts assigned to GOs “protein phosphorylation”, “defense response” and “ribosome biogenesis” are predominantly up-regulated in response to flooding with physical flow (Figure 3C). Among these, the expression changes in the transcripts related to “defense response” were more pronounced compared to those related to “protein phosphorylation” or “ribosome biogenesis” (Figure 4A). These results suggest that defense response pathways might be crucial for the regulation of plant response to flooding with physical flow. We also examined the overlap between transcripts that were specifically and significantly up-regulated in response to flooding with physical flow or submergence, as identified in this study, and those significantly up-regulated by various plant hormones in previous studies [49,50] (Figure 4B). Such meta-analysis is valuable for efficiently identifying plant hormone signals involved in pathways of interest from transcriptome data [51,52]. Our meta-analysis indicates that the proportion of transcripts responsive to methyl jasmonate (MJ) and salicylic acid (SA), hormones crucial for the defense responses [53], was higher among those up-regulated by flooding with physical flow compared to that in those up-regulated by submergence. These results support the idea that defense response pathways might play important roles in the response of plants to flooding with physical flow. Conversely, the proportion of transcripts responsive to abscisic acid (ABA), 1-aminocyclopropane-1-carboxylic Acid (ET precursor; ACC), brassinosteroids (BL), indole-3-acetic acid (AU; IAA) and gibberellic acid (GA) was lower among those specifically up-regulated by flooding with physical flow compared to those specifically up-regulated by submergence (Figure 4B). Furthermore, our meta-analysis demonstrated that the proportion of transcripts responsive to ABA, ACC, BL, cytokinin (CK) and IAA was higher among those specifically down-regulated by flooding with physical flow compared to those specifically down-regulated by submergence (Figure S4). Conversely, the proportion of transcripts responsive to MJ and SA was lower in those specifically down-regulated by flooding compared to those down-regulated exclusively by submergence. Plant hormones, such as IAA and GA have been well known to promote plant growth [54,55]. Down-regulation of transcripts involved in signaling of these plant hormones could be associated with growth inhibition of plants exposed to flooding accompanied by physical flow.

### 2.4. Up-Regulation of Transcripts Involved in JA or SA Synthesis and ROS Production

**Figure 5:**
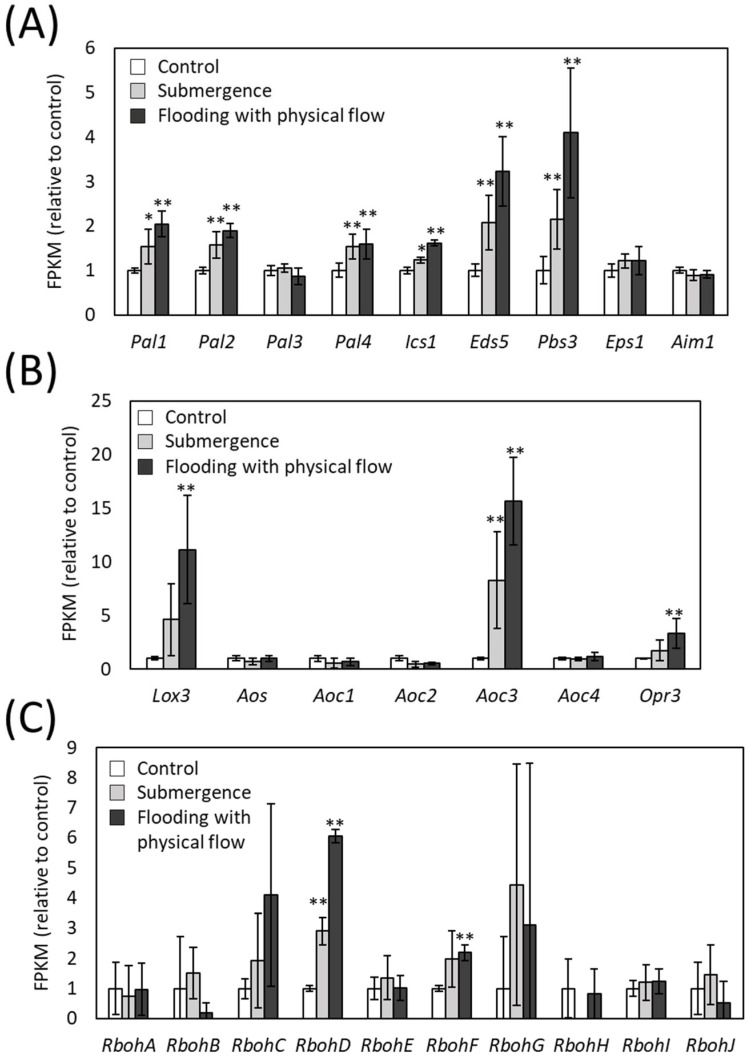
FPKM of transcripts involved in Salicylic acid (SA) synthesis (**A**), Jasmonic acid (JA) synthesis (**B**) or ROS production (**C**). Values relative to control are indicated. Error bars indicate standard deviation (*n* = 3). * and **: Student’s *t*-test significantly different at *p* < 0.05 and *p* < 0.01, respectively (compared to control).

To further confirm the involvement of defense response pathways in the responses of plants to flooding with physical flow, we analyzed the expression of transcripts essential for the synthesis of JA and SA in our transcriptome based on the fragments per kilobase of exon per million reads mapped (FPKM). Expression of key transcripts for SA synthesis was up-regulated by both submergence and flooding. Notably, up-regulation of *Pal1*, *Pal2*, *Ics1*, *Eds5* and *Pbs3* transcripts in response to flooding tended to be more pronounced compared to that in response to submergence (Figure 5A). Similar trend was also observed in the expression of JA synthesis transcripts, *Lox3*, *Aoc3* and *Opr3* (Figure 5B). These results indicate that JA and SA are involved in response of plants to both flooding with physical flow and submergence, but significance of these hormones in the response to flooding with physical flow might be more pronounced. As well as SA and JA, ROS also play pivotal roles in the regulation of defense response pathways. We therefore examined the expression of transcripts encoding Respiratory Burst Oxidase Homologues (RBOHs), ROS producing enzymes, in our transcriptome (Figure 5C). Expression of *RbohC*, *RbohD*, *RbohF* and *RbohG* showed a tendency to be up-regulated in response to both submergence and flooding. Especially, expression of *RbohD* was significantly up-regulated in response to both submergence and flooding, with higher expression under flooding. Similarly, significant up-regulation of *RbohF* expression was also observed under flooding. The roles of RBOHD and RBOHF, as well as SA and JA, in pathogen defense responses of plants have been thoroughly studied [32,33,34,53]. The results of our transcriptome analysis imply that defense responses regulated by the coordination between SA, JA and ROS signaling might play important roles in the response of plants to flooding.

Different transcripts encoding ROS scavenging enzymes are significantly up or down-regulated in response to submergence or flooding with physical flow (Figure S5). Four transcripts, *Cu*/*ZnSod1*, *Gr1*, *Gpx2* and *Aox3* were significantly up-regulated specifically in response to flooding with physical flow. In contrast, there are no ROS scavenging transcripts specifically down-regulated under this stress. No transcript was up-regulated specifically in response to submergence, while 5 transcripts *Cu*/*ZnSod3*, *Apx1*, *ApxS*, *Mdar2* and *Dhar3* were down-regulated specifically in response to this stress. Therefore, we can speculate that ROS level might be modulated by different mechanisms under submergence and flooding with physical flow.

### 2.5. Up-Regulation of AP2/Ethylene Response Factors in Response to Submergence and Flooding

**Figure 6:**
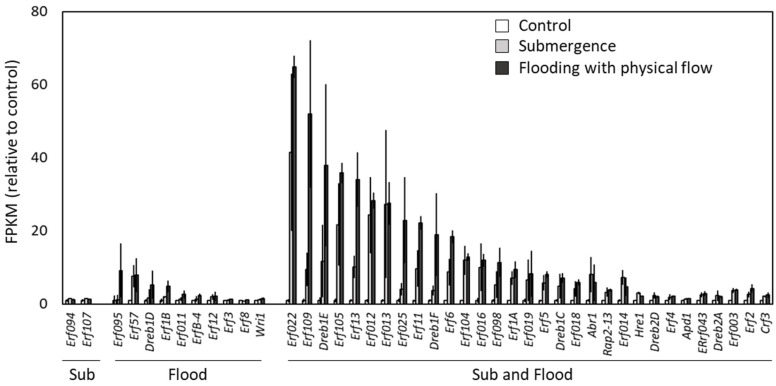
FPKM of transcripts encoding ERFs. Values relative to control are indicated. Error bars indicate standard deviation (*n* = 3). Transcripts were categorized into three groups; Sub: significantly up-regulated specifically in response to submergence, Flood: significantly up-regulated specifically in response to flooding with physical flow, Sub and Flood: significantly up-regulated in response to both stresses.

Previous studies have demonstrated the significance of ET signaling in the response of plants to anoxia including submergence [13]. To investigate if ET signaling is important for the response of plants to flooding with physical flow as well as submergence, we assessed the expression of transcripts encoding AP2/Ethylene Response Factors (ERFs) under submergence and flooding with physical flow (Figure 6). Among the *Erf* transcripts detected in our transcriptome analyses, the expression of 43 transcripts was significantly up-regulated in response to submergence, flooding or both. Of these, 31 transcripts were up-regulated in response to both submergence and flooding with physical flow. In addition, the expression of many of Erfs tended to be higher in plants exposed to flooding when compared to that in plants exposed to submergence, although ET-responsive transcripts were less represented in those specifically up-regulated during flooding compared to those up-regulated during submergence (Figure 4B). These results suggest that ET signaling plays important roles in the regulation of plant responses to flooding with physical flow as well as submergence. Under the conditions we tested, *Rap2.2* transcript, a homolog of rice *Sub1* gene [56], was not up-regulated in either submergence or flooding with physical flow, suggesting that the expression patterns of this transcript might vary depending on intensity or durations of stress.

### 2.6. Multiple Regression Analysis of Transcripts Involved in ROS Production, SA Synthesis and AU Signals

**Figure 7:**
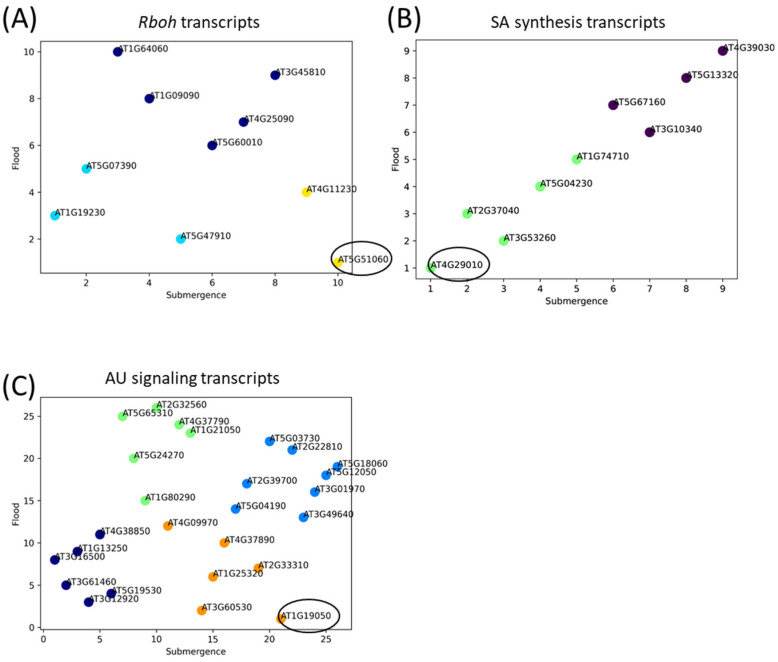
Multiple regression analysis of transcripts involved in ROS production ((**A**) *Rboh*s), SA (salicylic acid) synthesis (**B**) and AU (auxin) signaling (**C**). Circles indicate the transcript that showed the highest contribution to the determination of rosette diameter under flooding with physical flow. In this figure, the *X*- and *Y*-axis values indicate the ranking of transcripts that contribute to the effects on rosette diameter, with lower numbers indicating a higher contribution.

To investigate the links between rosette diameter and expression of transcripts involved in ROS production (*Rboh*s), SA synthesis and AU signals, we performed multiple regression analysis (Figure 7). In this analysis, rosette growth data were employed as the objective variable and transcript data as the explanatory variable to investigate how the growth data could be explained by the transcript data. The regression coefficient in the multiple regression analysis was used as an indicator of the influence of the growth data. We defined this index as “contribution value”. From the contribution value, we could evaluate the permutation of the transcript data that best explains the growth data. The contribution of each transcript to explaining the growth data under control, flooding with physical flow, and submergence conditions was estimated, and the contribution of each transcript under flooding and submergence conditions was represented in a scatterplot (Figure 7). According to this analysis, the expression of *RbohD* and *RbohC* transcripts, which were highly up-regulated in response to flooding with physical flow, showed a significant contribution to determining rosette diameter under these conditions (Figure 7A). The contribution of the *RbohC* transcript was particularly pronounced under flooding with a high flow rate (8 L/s). The *Aim1* transcript, involved in SA synthesis, exhibited a similar expression pattern to that of the *RbohC* transcript (Figure 7B). In contrast, the expression of the AU response transcript *Arr7* was down-regulated in response to both submergence and flooding with physical flow and demonstrated the highest contribution to determining rosette diameter under flooding with physical flow (Figure 7C).

We further investigated the expression of these transcripts that might be highly associated with inhibition of rosette growth in plants exposed to submergence or flooding with physical flow (Figure S6). For this analysis, the plants were subjected to submergence or flooding and sampled independently of RNA-Seq analysis. The expression of *RbohD* and *RbohC* transcripts tended to be up-regulated in response to flooding. Especially, the *RbohC* transcript demonstrated the highest expression in the plants exposed to flooding with a high flow rate (8 L/s). The *Aim1* transcript that is involved in the SA synthesis also exhibited a trend in the expression pattern similar to that of the *RbohC* transcript. In contrast, the expression of the AU response transcript, *Arr7* was down-regulated in response submergence and flooding.

### 2.7. Accumulation of H_2_O_2_ and Malondialdehyde in Plants Exposed to Submergence or Flooding with Physical Flow

**Figure 8:**
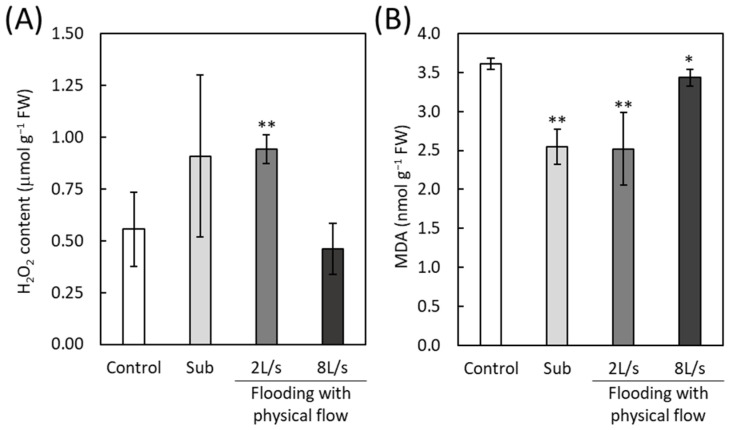
Accumulation of H_2_O_2_ (**A**) and MDA (**B**) in plants exposed to submergence (Sub) or flooding with different flow rates. Error bars indicate standard deviation (*n* = 3). * and **: Student’s *t*-test significantly different at *p* < 0.05 and *p* < 0.01, respectively (*n* = 3, compared to control).

The transcriptome data indicated that the modulation of the ROS level might be crucial for plant responses to submergence and flooding with physical flow. To investigate this, we measured H_2_O_2_ accumulation (Figure 8A). The H_2_O_2_ levels tended to increase in response to submergence and flooding with a low flow rate (2 L/s). Notably, a significant difference in H_2_O_2_ accumulation was observed between control plants and those exposed to flooding with a low flow rate. In contrast, plants exposed to flowing with a high flow rate (8 L/s) showed H_2_O_2_ accumulation levels similar to those of the control. To assess ROS-induced damage, we evaluated lipid peroxidation by measuring malondialdehyde (MDA) levels. Unlike H_2_O_2_, the MDA levels significantly decreased in response to submergence and flooding both with low and high flow rates (Figure 8B). In particular, plants exposed to flooding with a low flow rate or submergence showed a marked decrease in MDA levels.

## 3. Discussion

In this study, we demonstrated that flooding with physical flow impacts the rosette diameter of *Arabidopsis thaliana* plants. Notably, plants exposed to faster flow rates had smaller rosette diameters (Figure 2B). Our findings suggest two possibilities. First, flooding with physical flow may result in damage that negatively affects the growth of rosette leaves. Many studies have shown that multiple abiotic factors occurring simultaneously can exacerbate their negative effects on growth (Summarized in [57]). Thus, the combined effects of submergence and physical flow could inhibit leaf growth. Alternatively, smaller rosette leaves might represent an adaptive response to physical flow. Alterations in growth and development are known to be strategies for plants to adapt to abiotic stresses. For example, leaf anatomical changes in response to drought are associated with modulation of physiological processes, such as transpiration and photosynthesis [58]. In addition, altered plant morphology can lower leaf temperature under high-temperature conditions [59]. In our study, we speculate that smaller rosette leaves might help to prevent damage from the “shearing” effects of physical flow. This speculation could be further supported by findings that rosette diameter in plants is associated with the expression of *Rboh* transcripts as well as hormone signaling transcripts (Figure 7). ROS produced by RBOH enzymes are involved in many beneficial processes, including the regulation of growth and responses to biotic and abiotic stresses [31]. Furthermore, the MDA level significantly decreased in response to all stress conditions we tested despite the increase in H_2_O_2_ level under submergence and flooding at a low flow rate (Figure 8). H_2_O_2_ appears to be tightly regulated under these conditions depending on the flow rate. However, under the condition we tested, changes in H_2_O_2_ do not result in oxidative damage to cells. This finding also supports the hypothesis that modulating ROS as signaling molecules is essential for the response of plants to these conditions. Moreover, down-regulation of transcripts associated with signaling of plant hormones that are involved in the regulation of growth, such as auxin, could also be associated with growth inhibition of plants exposed to flooding accompanied by physical flow (Figure 7). Therefore, it is essential to investigate how RBOH-dependent ROS and plant hormone signaling might regulate plant growth under flooding with physical flow. In addition, examining the response of mutants deficient in RBOH enzymes or hormone signaling to flooding with physical flow could provide valuable insights. Although the role of RBOHC in root hair development has been extensively studied [60], its involvement in waterlogging responses has not been addressed. Our results suggest potential new roles for RBOHC in plant stress responses.

While ROS regulation may be associated with the growth response of plants to flooding with physical flow, H_2_O_2_ accumulation is not directly linked to rosette diameter (Figure 2B and Figure 8A). The H_2_O_2_ levels increased in response to submergence and flooding with a low flow rate but remained unchanged under flooding with a high flow rate (Figure 8A). We can speculate that certain ROS scavenging mechanisms are specifically activated by flooding with a high flow rate to finely modulate the ROS accumulation. In this study, we found that several transcripts encoding ROS scavenging enzymes were up-regulated in response to flooding with a high flow rate (Figure S5). It is also plausible that specific ROS-dependent signals, activated in specific tissues/organs, rather than overall ROS accumulation, are key in regulating plant growth responses to flooding with physical flow. Spatial patterns of ROS accumulation and signaling have been shown to be altered by abiotic stresses [61,62]. In addition, it has been suggested that each RBOH plays distinct roles in developmental processes across different tissues and in stress responses [31]. Thus, future studies dissecting ROS accumulation and its regulatory pathways in different individual leaves and the shoot apical meristem may provide valuable insights into the detailed mechanisms of ROS signaling in response to submergence and physical flow. Another possibility is that other signals function together with ROS regulatory systems. In this study, we also demonstrated the potential involvement of pathogen defense mechanisms in the regulation of plant responses to flooding with physical flow (Figure 3C, Figure 4 and Figure 5). *RbohD* and *RbohF* transcripts, as well as those associated with SA and JA signaling, were significantly up-regulated in response to both submergence and flooding with physical flow, with a more pronounced up-regulation under flooding with physical flow (Figure 5). Key players of pathogen defense mechanisms have been implicated in plant responses to waterlogging, as hypoxic conditions caused by waterlogging favor fungal and bacterial pathogens [24,63]. Indeed, it was demonstrated that RBOHD regulates the expression of hypoxia induced genes, and modulation of the H_2_O_2_ level by ET is associated with RBOH functions [35]. Furthermore, SA and JA, which are involved in responses to biotrophic and necrotrophic pathogens, respectively, increase in response to waterlogging [24]. SA promotes apoptosis necessary for aerenchyma formation and adventitious root development in soybeans [24]. JA positively influences waterlogging tolerance by increasing ET concentration in conifers [24] but can also suppress root growth and SA activity in soybeans [24]. Although previous studies demonstrated the involvement of key players of pathogen defense in the response of plants to waterlogging, it should be noted that our study is the first to analyze the mechanisms underlying the response of plants to flooding with “physical flow”. To specifically tailor signals for the adaptation to flooding with physical flow, transcripts involved in pathogen defense specifically up-regulated by this condition (Table S8) might be integrated with SA- and JA-dependent pathways as well as functions of RBOHs. It is therefore crucial to investigate the roles of these flooding-specific transcripts and their integration with major pathogen defense mechanisms. Furthermore, exploring the integration of pathogen defense mechanisms with other signals, such as those associated with protein phosphorylation and ribosome biogenesis (GO terms represented in transcripts up-regulated by flooding with physical flow), would be of significant interest.

Moreover, the integration of Ca^2+^ signaling with RBOH-dependent ROS signaling and pathogen responses has been extensively studied [31,34,38,64]. For instance, both ROS and Ca^2+^ are required for the activation of hypersensitive responses to pathogens, which are accompanied by programmed cell death [64]. Moreover, the binding of Ca^2+^ to EF-hand motifs is crucial for the activation of RBOH proteins, highlighting the tight linkage between Ca^2+^ and ROS signaling [65]. Thus, exploring the integration of Ca^2+^ signaling with the mechanisms underlying plant responses to submergence and flooding with physical flow should be a focus of future studies.

AP2/ERFs are known to play key roles in the regulation of plant responses to biotic and abiotic stresses [66]. In this study, we found that 10 AP2/ERFs were specifically up-regulated in response to flooding with physical flow (Figure 6). Among these, the *Erf095* transcript exhibited the highest increase. A previous study demonstrated that ERF095 positively regulates heat tolerance of Arabidopsis thanliana via activation of Heat Shock Transcription Factor A2 (HSFA2) [67]. *Dreb1D* also showed dramatic up-regulation in response to flooding with physical flow. DREB1D was shown to inhibit ABA-dependent drought response by negatively regulating Xerico, a stress-responsive RING E ubiquitin ligase [68]. These finding suggest that the AP2/ERF-dependent plant response to flooding with physical flow at least partially overlaps with response mechanisms to other abiotic stresses. In addition, *Erf011* and *Erf8*, which are involved in pathogen defense [69,70], were also specifically up-regulated in response to flooding with physical flow, supporting the significance of pathogen defense mechanisms in response of *Arabidopsis thaliana* to this condition. However, the roles of these 10 AP2/ERFs specifically up-regulated in response to flooding with physical flow in the regulation of plant responses to hypoxia have not been reported, indicating that specific ethylene signaling involving these AP2/ERFs operates under flooding with physical flow. In this study, *Hre1*, also known as *Erf73*, was up-regulated in response to both submergence and flooding with physical flow. It has been demonstrated that RBOHD-dependent regulation of ethylene signaling may be mediated by ERF73 under hypoxia [71]. The expression of *Erf73* transcript was inhibited in the double mutant of *Arabidopsis thaliana* deficient in RBOHD and Ethylene-Insensitive protein 2 (EIN2) under hypoxia [71]. In addition, *Dreb2A*, a key regulator of abiotic stress responses, was also up-regulated by both stress conditions in this study. While the negative regulator of drought response, *Dreb1D*, was specifically up-regulated in response to flooding with physical flow, the positive regulator of drought, *Dreb2A*, was also up-regulated in response to both flooding with physical flow and submergence. These finding suggest that the AP2/ERF-dependent response of *Arabidopsis thaliana* to flooding with physical flow is intertwined with response mechanisms to drought-related stresses. It would be interesting to explore how AP2/ERF-dependent drought response mechanisms are modulated during flooding with physical flow.

In this study, transcripts associated with GO terms “plasma membrane” and “plant-type cell wall” were highly represented among those up-regulated specifically by flooding with physical flow, as well as those up-regulated specifically by submergence (Tables S6 and S8). The significance of mechanisms operating at the plasma membrane or apoplast in the regulation of plant responses to hypoxia has been indicated in previous studies. Plasma membrane proteome analyses in maize roots under hypoxia revealed alterations in redox systems and increased accumulation of aquaporins [72]. Furthermore, Zelinová and colleagues compared two barley cultivars in their response to hypoxia [73]. While the hypoxia-sensitive cultivar showed root growth arrest accompanied by increased lipid peroxidation and cell death, the hypoxia-tolerant cultivar exhibited increased apoplastic nitric oxide in the root tips. Moreover, the redox activity of the root tip was maintained at a higher level in the tolerant cultivar compared to the sensitive one under prolonged hypoxia. These findings underscore the integration between mechanisms operating in the plasma membrane and redox regulatory systems. Building upon these findings and our results, it can be speculated that to adapt to flooding with physical flow, mechanisms operating in the plasma membrane or apoplast might integrate with ROS signals regulated by RBOH proteins, which are localized in the plasma membrane [31], as well as pathogen defense mechanisms specifically activated by flooding with physical flow. However, it should be noted that previous studies primarily focused on roots, highlighting the need to investigate how membrane-associated mechanisms integrate with pathogen defense mechanisms and ROS regulatory systems in shoots under flooding with physical flow. In a recent study, it has been demonstrated that waterlogging can activate long-distance signaling regulated by RBOHD-dependent ROS [39]. This propagation of long-distance signaling is known to be associated with the functions of plasmodesmata proteins [74]. Therefore, integration between signals activated in apoplast, plasma membrane and plasmodesmata could also be of interest for future studies.

This study represents the first exploration of plant responses to flooding with physical flow. We revealed the potential involvement of pathogen defense mechanisms, regulated by RBOH-dependent ROS, as well as JA and SA, in regulating plant responses to flooding with physical flow. As this research topic is still in its initial stages, further studies, including analyses of mutants deficient in RBOHs, SA signaling, or AU signaling, are necessary to elucidate the detailed mechanisms underlying plant responses to flooding with physical flow. In addition, future studies should analyze other parameters such as pathogen resistance. It would also be valuable to investigate how flooding with physical flow affects the growth and physiological processes of other plant species and different Arabidopsis accessions.

This study suggests a new type of interdisciplinary research between plant biology and engineering. Further advances could open new avenues for research focusing on the social impacts of flooding from both biological and engineering perspectives.

## 4. Materials and Methods

### 4.1. Plant Material and Growth Conditions

Wild-type (WT) *Arabidopsis thaliana* (ecotype Columbia-0) was grown on a peat pellet under controlled conditions: 21–24 °C, a 16 h light cycle, 50 µmol m^−2^s^−1^, and approximately 50–70% humidity on the steel shelf equipped with light bulbs. The plants were watered with tap water. Despite the absence of nutrient supplementation during growth, no growth retardation or symptoms of nutrient deficiency were observed (Figure S7). In this study, healthy plants of uniform size were selected for the experiments.

### 4.2. Stress Treatment and Measurement of Growth Parameters

Ten *Arabidopsis thaliana* plants grown on peat pellet for approximately 4 weeks were fixed by using metal anchors on the open water channel measuring 50 cm in width and 10 m in length, which can artificially create water flow in one direction (Figure 1 and Figure S1). In this system, water inflow was applied from one side, with an equal amount of outflow from the opposite side. The flow rate was monitored by flow velocimeters with propeller of 1 cm diameter. We adjusted the flow rate to 2 L/s and 8 L/s, which is equivalent to 5 cm/s and 30 cm/s, respectively. To modulate the water depth, the weir height at the downstream end of the water channel was adjusted (Figure 1B and Figure S1D,E). In addition, the plants were also subjected to submergence in water with 10 cm depth without physical flow in parallel in a plastic container. Following the exposure to these stresses for 30 min, the number of leaves and rosette diameter were scored from 1 day following the application of stresses. The length of inflorescent stem was measured from 6th day following the application of stresses.

### 4.3. RNA-Seq Analysis and Quantitative Real-Time PCR

Total RNA was extracted by using TRIzol reagent (Thermo Fisher Scientific, Waltham, MA, USA) from three independent biological replicates, each pooled from fresh rosette of 10 plants that were frozen in liquid nitrogen immediately after the sampling. RNA purification, RNA sequence library preparation, sequencing, mapping, and gene expression analysis were performed by K.K. DNAFORM (Yokohama, Kanagawa, Japan). The qualities of total RNA were assessed by Bioanalyzer (Agilent) to ensure that RIN (RNA integrity number) was over 7.0. After poly(A) + RNA enrichment by NEBNext Poly(A) mRNA Magnetic Isolation Module (New England BioLabs, Ipswich, MA, USA), double-stranded cDNA libraries (RNA-seq libraries) were prepared using SMARTer stranded RNA-Seq kit v2 (Clontech, Mountain View, CA, USA) according to the manufacturer’s instructions. RNA-seq libraries were sequenced using paired end reads (50 nt of read1 and 25 nt of read2) on a NextSeq 500 instrument (Illumina, San Diego, CA, USA). The raw reads obtained were trimmed and quality-filtered using the Trim Galore! (version 0.4.4), Trimmomatic (version 0.36), and cutadapt (version 1.16) software. Trimmed reads were then mapped to the Arabidopsis_thaliana TAIR10.49 using STAR (version 2.7.2b). Reads on annotated genes were counted using featureCounts (version 1.6.1). FPKM values were calculated from mapped reads by normalizing to total counts and transcript. Differentially expressed genes were detected using the DESeq2 package (version 1.20.0). The list of differentially expressed genes detected by DESeq2 (basemean > 5 and *p*-adj < 0.05) was used for GO enrichment analysis, as described below. The data of the transcriptome analysis were uploaded to Gene Expression Omnibus (Accession number: GSE268967). First-strand complementary DNA (cDNA) was produced after DNAseI treatment from 1 mg of total RNA with M-MuLV reverse transcriptase (New England Biolabs). The accumulation of transcripts was analyzed by quantitative real-time polymerase chain reaction (qPCR) with the Quant Studio 3 system (Thermo Fisher Scientific, Waltham, MA, USA) and Thunderbird qPCR Master Mix (Toyobo, Tokyo, Japan). Threshold cycle values for target genes were calculated with the cycle threshold of Ef1-α as an internal control. The primer pairs used for qPCR are shown in Table S1.

### 4.4. Measurement of H_2_O_2_ and MDA Contents

H_2_O_2_ measurement was performed using Amplex Red reagent Red (Molecular Probes, Invitrogen, Waltham, MA, USA, http://www.invitrogen.com/ (accessed on 31 October 2024)), as previously described [75]. Malondialdehyde (MDA) was measured as previously described using an extinction coefficient for MDA of 155 mM^−1^cm^−1^ [75].

### 4.5. Database and Statistical Analyses

Transcripts significantly up- or down-regulated in response to submergence or flooding with physical flow were analyzed by the Gene Ontology (GO) database, DAVID (https://david.ncifcrf.gov/tools.jsp, accessed on 30 August 2023). The overlaps between transcripts significantly up-regulated in response to each of the different stresses and those up-regulated in response to different plant hormones [49,50] were determined as previously described [51]. Differences were discriminated by a one-tailed Student’s *t*-test. Multiple regression analysis was performed using the Python package “statsmodels”. The version of python is 3.9.

## Data Availability

Other data will be made available on request.

## Supplementary Materials

The following supporting information can be downloaded at: https://www.mdpi.com/article/10.3390/plants13243508/s1, Figure S1: The channel used in this study. (A,B) Appearance of the channel from side (A) and top (B). (C) Inside of the channel. (D) side and (E) top view of plants treated with flooding, which allow us to indicate the direction of water flow; Figure S2: MA-plot visualizing the differentially expressed transcripts between control and submergence (A) or control and flooding (B); Figure S3: Characteristics of transcripts down-regulated in response to flooding with physical flow. (A) Venn diagram showing the overlap between transcripts down-regulated in response to submergence or flooding. (B) GO terms of biological processes represented in transcripts specifically down-regulated in response to flooding; Figure S4: Representation of hormone response transcripts among the transcripts specifically down-regulated in response to submergence or flooding; Figure S5: FPKM of transcripts encoding ROS scavenging enzymes. Values relative to control are indicated. Bars indicate standard deviation (*n* = 3). Transcripts significantly up- or down-regulated in response to submergence, flooding with physical flow or both are indicated in the table (lower panel); Figure S6: Expression of transcripts involved in ROS production, SA synthesis and AU signaling in plants exposed to submergence and flooding with different flow rates. Ctrl: Control, Sub: Submergence; Figure S7: Four-week-old *Arabidopsis thaliana* plants used for the experiments; Table S1: Mapping information of RNA-Seq analysis; Table S2: Transcripts specifically up-regulated in response to submergence; Table S3: Transcripts specifically up-regulated in response to flooding with physical flow; Table S4: Transcripts commonly up-regulated in response to submergence and flooding with physical flow; Table S5: GO terms significantly represented in the transcripts that were specifically up-regulated in response to submergence; Table S6: Transcripts that are assigned to GOs listed in Table S5; Table S7: GO terms significantly represented in the transcripts that were specifically up-regulated in response to flooding with physical flow; Table S8: Transcripts belong to GOs listed in Table S7; Table S9: GO terms significantly represented in the transcripts that were commonly up-regulated in response to submergence and flooding with physical flow; Table S10: Transcripts specifically down-regulated in response to submergence; Table S11: Transcripts specifically down-regulated in response to flooding with physical flow; Table S12: Transcripts commonly down-regulated in response to submergence and flooding with physical flow; Table S13: GO terms significantly represented in the transcripts that were specifically down-regulated in response to submergence; Table S14: Transcripts belong to GOs listed in Table S13; Table S15: GO terms significantly represented in the transcripts that were specifically down-regulated in response to flooding with physical flow; Table S16: Transcripts belong to GOs listed in Table S15; Table S17: GO terms significantly represented in the transcripts that were commonly down-regulated in response to submergence and flooding with physical flow; Table S18: Primers used for qRT-PCR analysis.
