# Supplementary material for: Response of Arabidopsis thaliana to Flooding with Physical Flow
Source: Plants (Basel). 2024 Dec 16;13(24):3508. doi: 10.3390/plants13243508 (PMC11678080; doi:10.3390/plants13243508)
Supplement: Supplementary file 1 [file plants-13-03508-s001.zip › Supplemental Figures.pptx]

## Slide 1
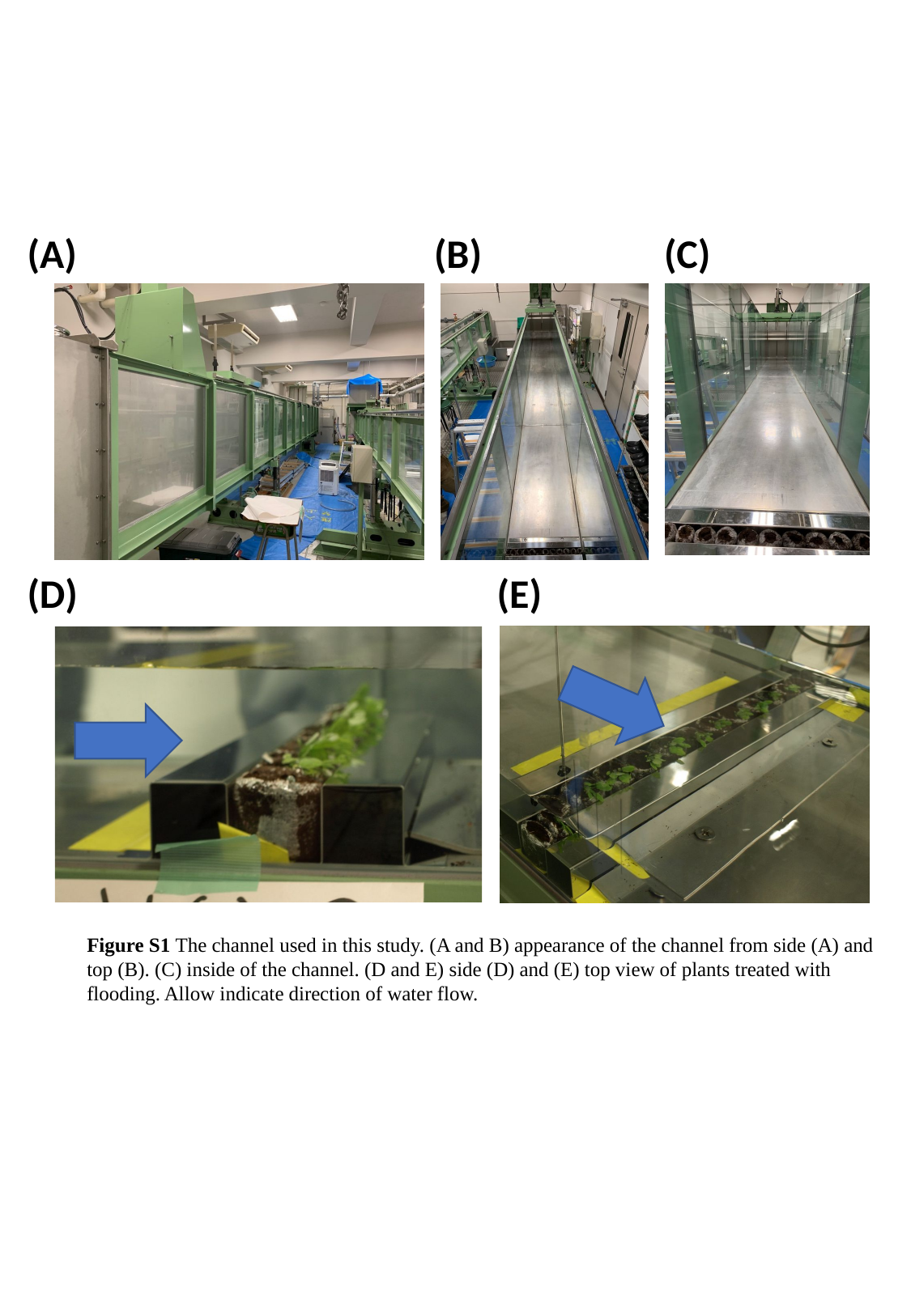

(B) (C)
(D) (E)
Figure S1 The channel used in this study. (A and B) appearance of the channel from side (A) and top (B). (C) inside of the channel. (D and E) side (D) and (E) top view of plants treated with flooding. Allow indicate direction of water flow.

## Slide 2
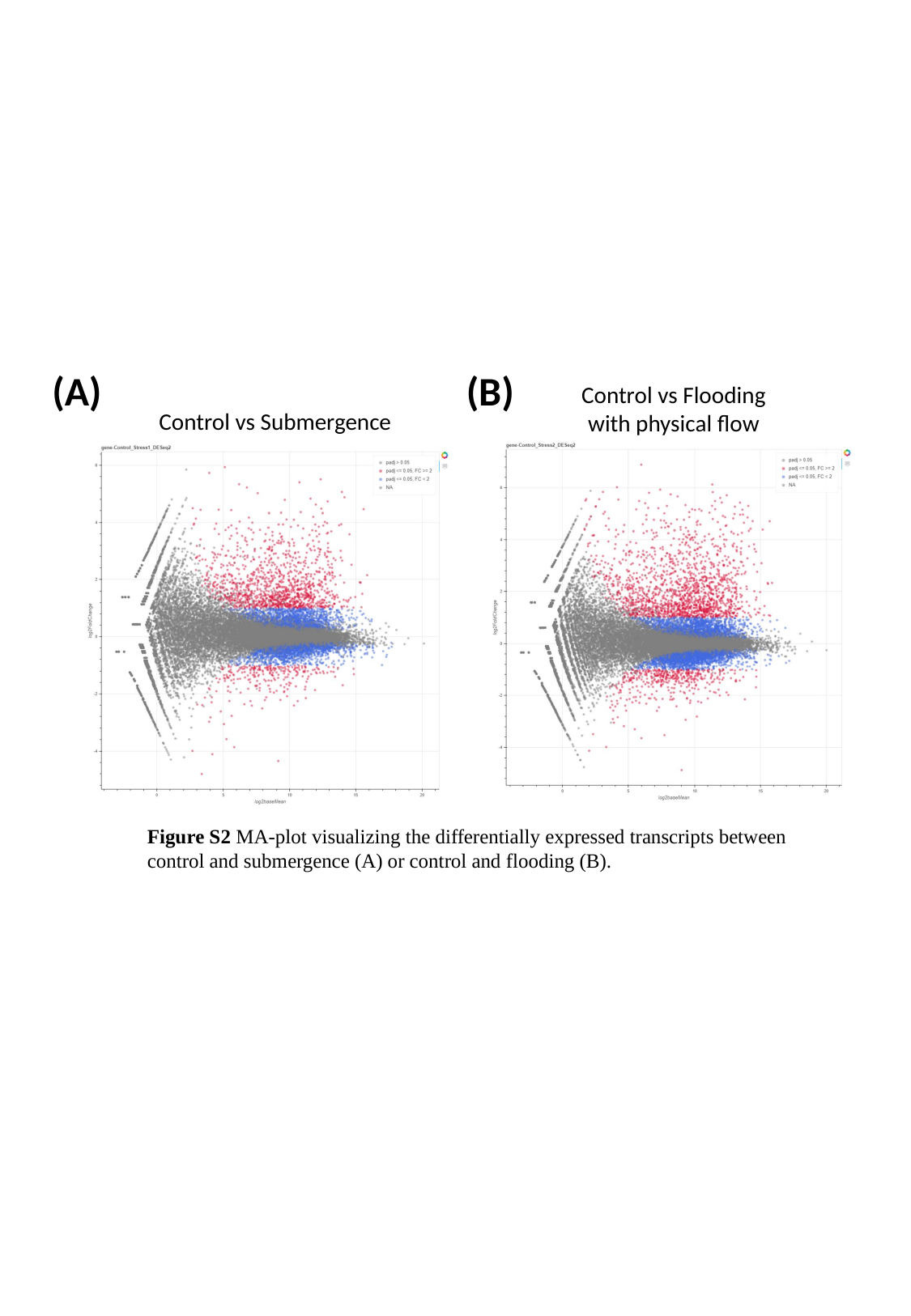

(A) (B)
Control vs Flooding with physical flow
Control vs Submergence
Figure S2 MA-plot visualizing the differentially expressed transcripts between control and submergence (A) or control and flooding (B).

## Slide 3
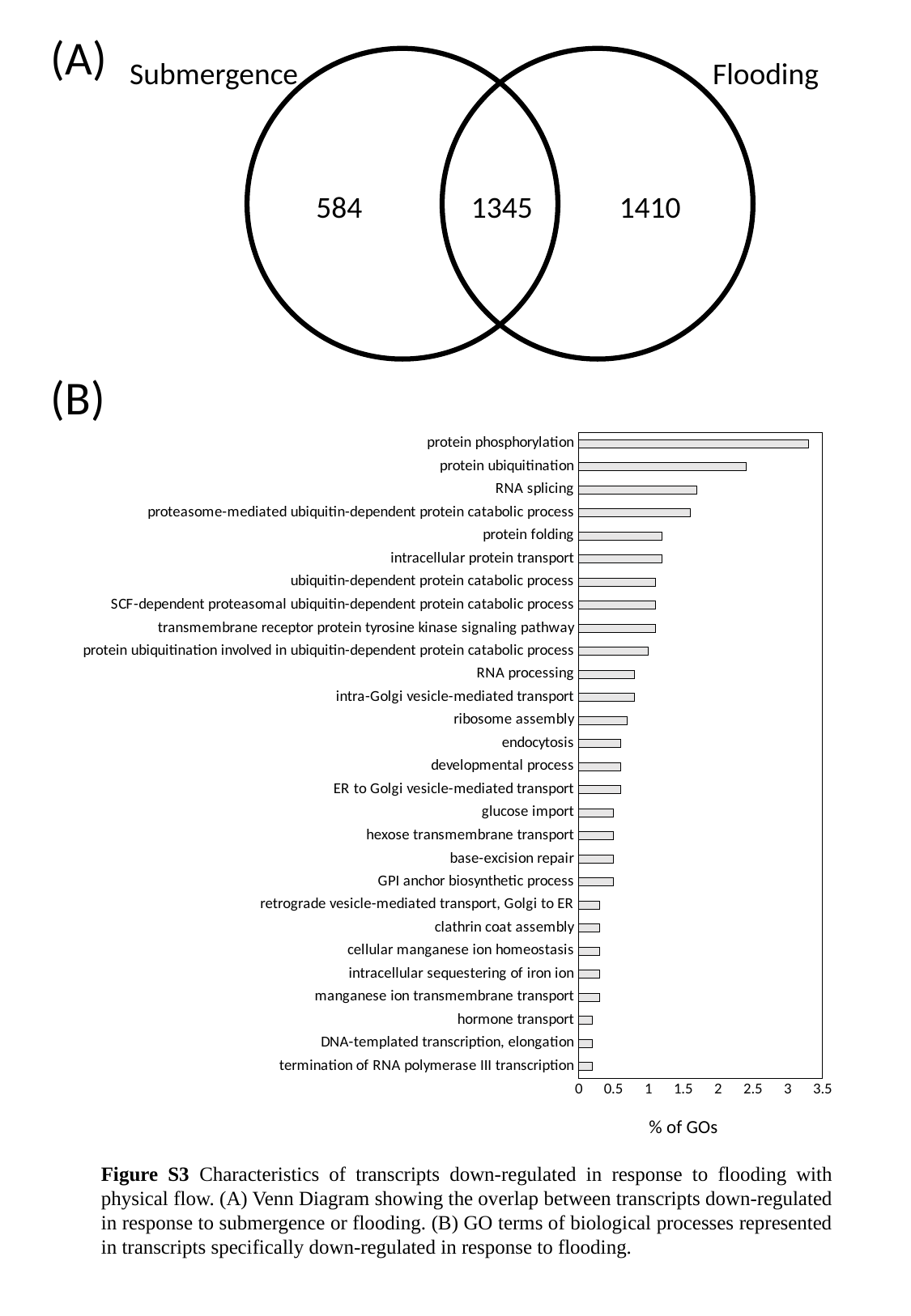

(A)
(B)
Submergence
Flooding
584
1345
1410
### Chart
| Category | |
|---|---|
| termination of RNA polymerase III transcription | 0.2 |
| DNA-templated transcription, elongation | 0.2 |
| hormone transport | 0.2 |
| manganese ion transmembrane transport | 0.3 |
| intracellular sequestering of iron ion | 0.3 |
| cellular manganese ion homeostasis | 0.3 |
| clathrin coat assembly | 0.3 |
| retrograde vesicle-mediated transport, Golgi to ER | 0.3 |
| GPI anchor biosynthetic process | 0.5 |
| base-excision repair | 0.5 |
| hexose transmembrane transport | 0.5 |
| glucose import | 0.5 |
| ER to Golgi vesicle-mediated transport | 0.6 |
| developmental process | 0.6 |
| endocytosis | 0.6 |
| ribosome assembly | 0.7 |
| intra-Golgi vesicle-mediated transport | 0.8 |
| RNA processing | 0.8 |
| protein ubiquitination involved in ubiquitin-dependent protein catabolic process | 1.0 |
| transmembrane receptor protein tyrosine kinase signaling pathway | 1.1 |
| SCF-dependent proteasomal ubiquitin-dependent protein catabolic process | 1.1 |
| ubiquitin-dependent protein catabolic process | 1.1 |
| intracellular protein transport | 1.2 |
| protein folding | 1.2 |
| proteasome-mediated ubiquitin-dependent protein catabolic process | 1.6 |
| RNA splicing | 1.7 |
| protein ubiquitination | 2.4 |
| protein phosphorylation | 3.3 |% of GOs
Figure S3 Characteristics of transcripts down-regulated in response to flooding with physical flow. (A) Venn Diagram showing the overlap between transcripts down-regulated in response to submergence or flooding. (B) GO terms of biological processes represented in transcripts specifically down-regulated in response to flooding.

## Slide 4
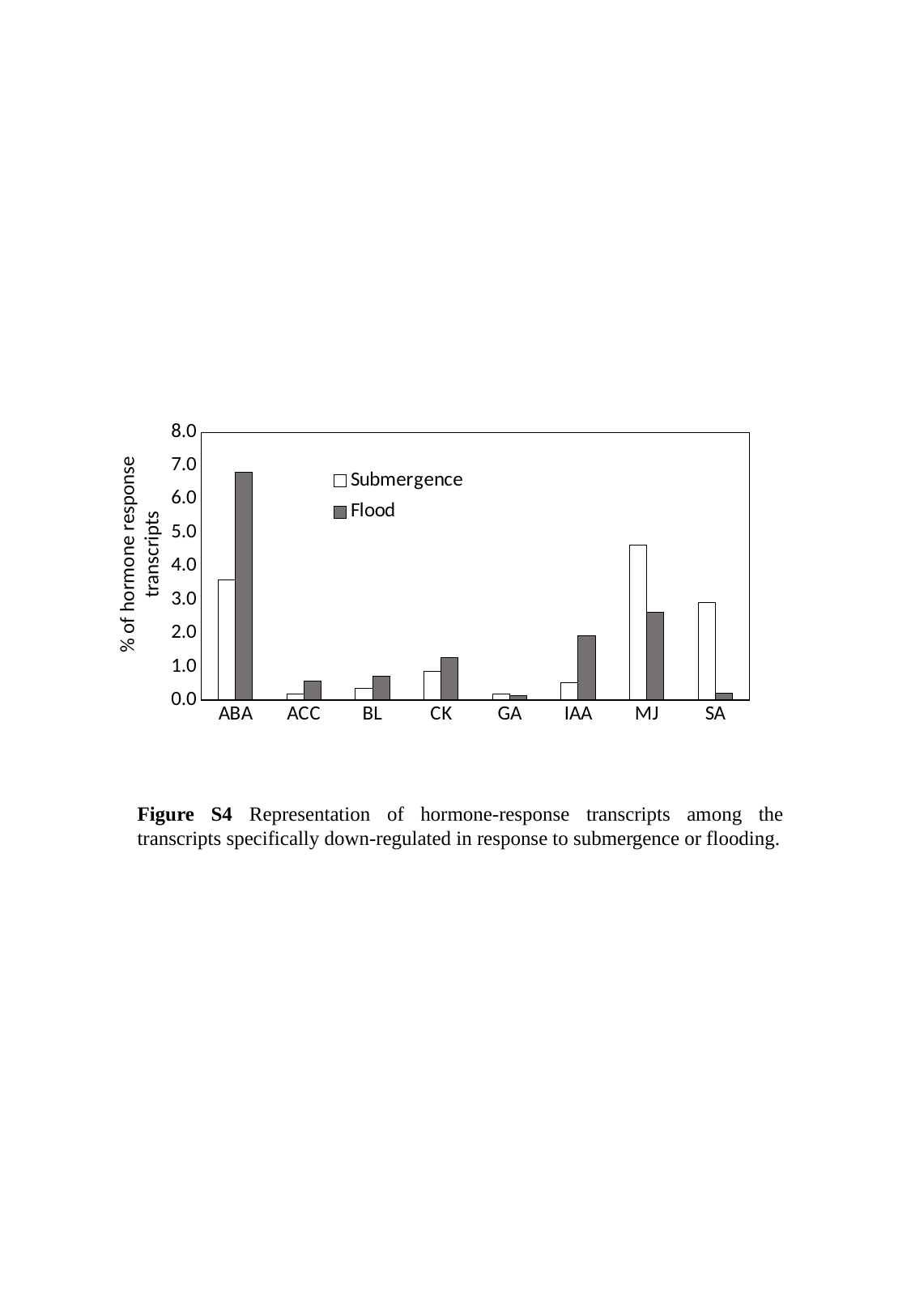

### Chart
| Category | Submergence | Flood |
|---|---|---|
| ABA | 3.595890410958904 | 6.808510638297872 |
| ACC | 0.17123287671232876 | 0.5673758865248227 |
| BL | 0.3424657534246575 | 0.7092198581560284 |
| CK | 0.8561643835616438 | 1.276595744680851 |
| GA | 0.17123287671232876 | 0.14184397163120568 |
| IAA | 0.5136986301369862 | 1.9148936170212765 |
| MJ | 4.623287671232877 | 2.624113475177305 |
| SA | 2.910958904109589 | 0.2127659574468085 |% of hormone response transcripts
Figure S4 Representation of hormone-response transcripts among the transcripts specifically down-regulated in response to submergence or flooding.

## Slide 5
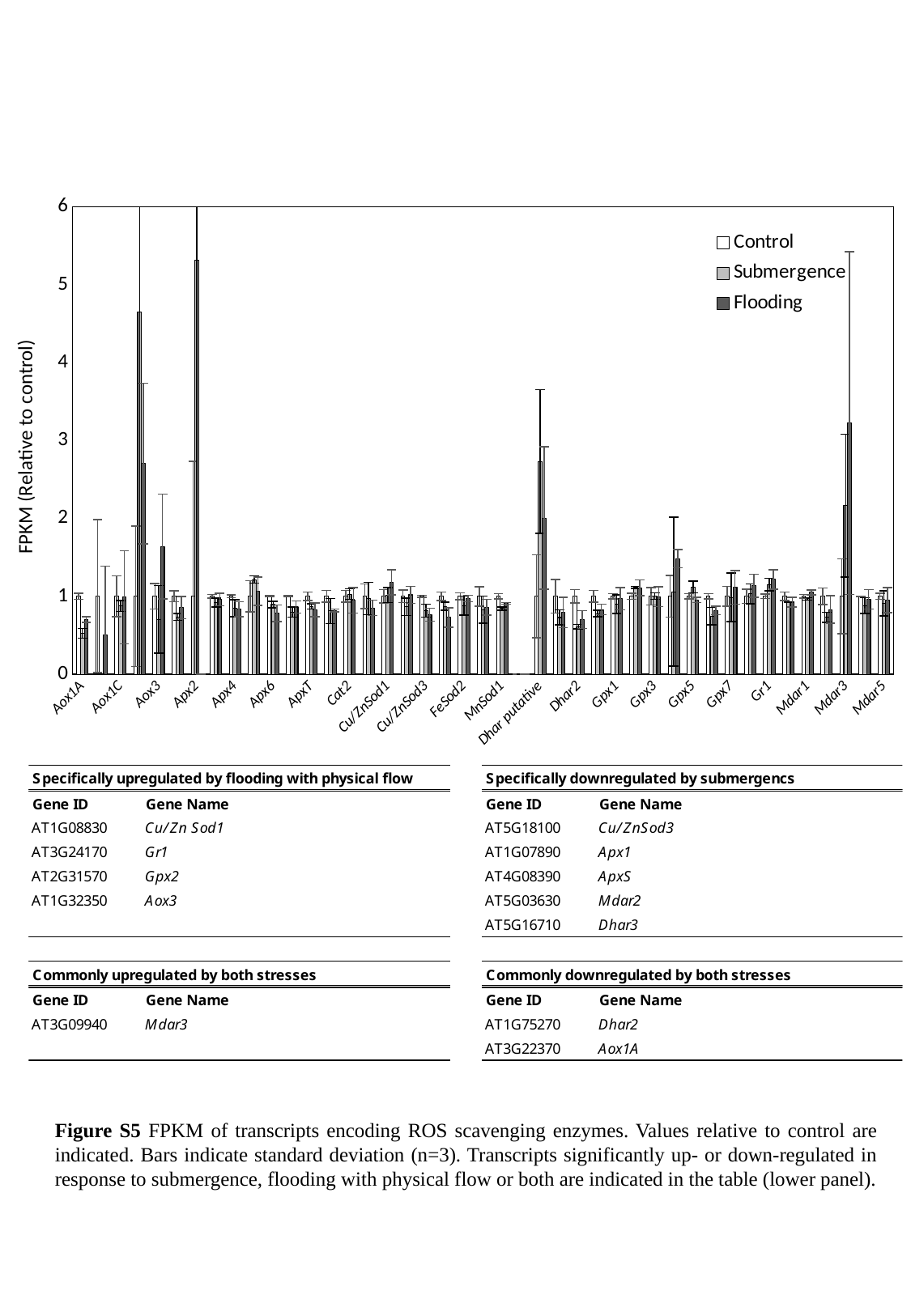

### Chart
| Category | Control | Submergence | Flooding |
|---|---|---|---|
| Aox1A | 1.0 | 0.5229790369433027 | 0.7024406158086579 |
| Aox1B | 1.0 | 0.0 | 0.5085844156588374 |
| Aox1C | 1.0000000000000002 | 0.8757957961252664 | 0.9872886705289985 |
| Aox2 | 1.0 | 4.654022196246866 | 2.7051204380415093 |
| Aox3 | 0.9999999999999999 | 0.7048738018074944 | 1.6378920365448038 |
| Apx1 | 1.0000000000000002 | 0.7365904899027954 | 0.8552639191370558 |
| Apx2 | 1.0 | 5.318355264387675 | 0.0 |
| Apx3 | 0.9999999999999999 | 0.9200511277792138 | 0.9593475594832266 |
| Apx4 | 1.0000000000000002 | 0.8455487117653878 | 0.8341559391893544 |
| Apx5 | 1.0 | 1.213365735144636 | 1.0631504697139778 |
| Apx6 | 1.0 | 0.8937973532254168 | 0.7826012808983093 |
| ApxS | 1.0 | 0.7961694243412523 | 0.8625893442380826 |
| ApxT | 0.9999999999999999 | 0.8706510895182609 | 0.8251911270329826 |
| Cat1 | 1.0 | 0.8109501417769923 | 0.8165789679438569 |
| Cat2 | 0.9999999999999999 | 1.0277345806851872 | 0.9474735790032085 |
| Cat3 | 1.0 | 0.9722088222180508 | 0.8514105849309176 |
| Cu/ZnSod1 | 1.0 | 1.015896738650085 | 1.179643248603252 |
| Cu/ZnSod2 | 1.0 | 0.8637699437766367 | 1.0180785098825715 |
| Cu/ZnSod3 | 1.0 | 0.8154820845595903 | 0.7630297368379998 |
| FeSod1 | 1.0 | 0.870917559077514 | 0.727941147727052 |
| FeSod2 | 1.0 | 0.8796027141061397 | 0.9720123205289662 |
| FeSod3 | 1.0 | 0.8299778614591345 | 0.8604231052515345 |
| MnSod1 | 1.0 | 0.8438062625524901 | 0.9103305939000962 |
| MnSod2 | 0.0 | 0.0 | 0.0 |
| Dhar putative | 1.0 | 2.7305935813908935 | 2.004646601265306 |
| Dhar1 | 1.0000000000000002 | 0.7275455644525651 | 0.7924514468473274 |
| Dhar2 | 1.0 | 0.6110211835393923 | 0.7031174024510651 |
| Dhar3 | 1.0 | 0.7779370699254993 | 0.8296623381780411 |
| Gpx1 | 1.0 | 0.897411986056689 | 0.9701648541081719 |
| Gpx2 | 1.0 | 1.1140104918903175 | 1.110213676885705 |
| Gpx3 | 1.0000000000000002 | 1.0015637605158343 | 0.9939444153954944 |
| Gpx4 | 1.0 | 1.0583037293997262 | 1.481851801951277 |
| Gpx5 | 1.0 | 1.1201494414345536 | 0.9527250756750104 |
| Gpx6 | 1.0 | 0.7451364540890433 | 0.819112040048493 |
| Gpx7 | 1.0 | 0.9865047418590734 | 1.1147238502390948 |
| Gpx8 | 1.0 | 1.0303258496772505 | 1.1330543415251058 |
| Gr1 | 1.0 | 1.1501645675813383 | 1.2166187907211052 |
| Gr2 | 1.0 | 0.9250129348550195 | 0.9270112032859715 |
| Mdar1 | 1.0 | 0.9665578466092546 | 1.0504403388831638 |
| Mdar2 | 0.9999999999999999 | 0.7304028646768064 | 0.8304824123409524 |
| Mdar3 | 1.0 | 2.1620464923785927 | 3.2239296127333392 |
| Mdar4 | 1.0 | 0.8806629855123523 | 0.9608557140212204 |
| Mdar5 | 1.0000000000000002 | 0.9086679789935194 | 0.9491133267174812 |FPKM (Relative to control)
Figure S5 FPKM of transcripts encoding ROS scavenging enzymes. Values relative to control are indicated. Bars indicate standard deviation (n=3). Transcripts significantly up- or down-regulated in response to submergence, flooding with physical flow or both are indicated in the table (lower panel).

## Slide 6
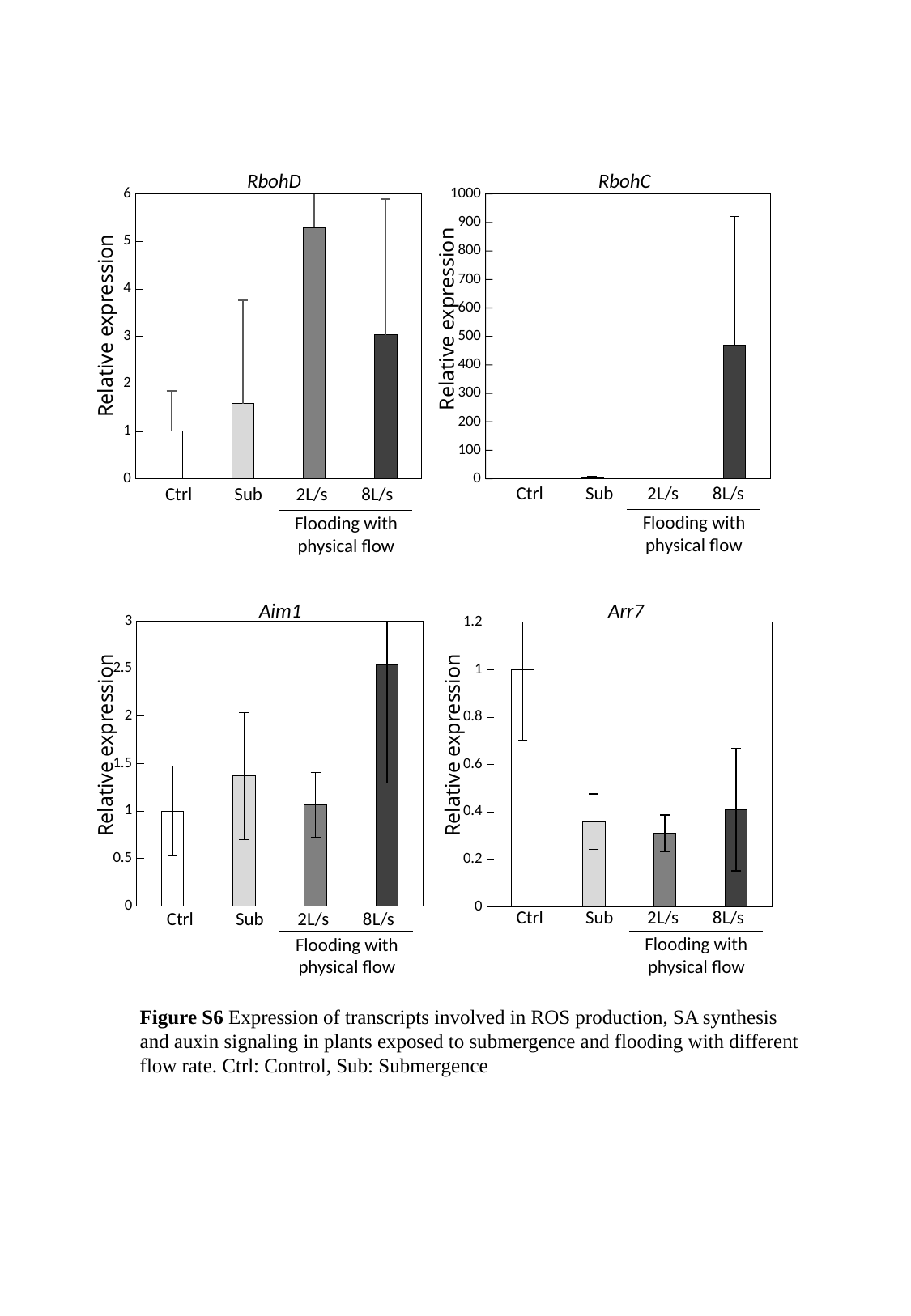

RbohD
RbohC
### Chart
| Category | |
|---|---|
| Ctrl | 1.0 |
| Sub | 5.052817261248372 |
| 2L/s | 1.2130248469453764 |
| 8L/s | 468.687825350605 |
### Chart
| Category | |
|---|---|
| contorol | 1.0 |
| 0 | 1.5869061047709818 |
| 2 | 5.291849142824844 |
| 7.9 | 3.0373929677747094 |Relative expression
Relative expression
Ctrl Sub 2L/s 8L/s
Ctrl Sub 2L/s 8L/s
Flooding with physical flow
Flooding with physical flow
Aim1
Arr7
### Chart
| Category | |
|---|---|
| Ctrl | 1.0 |
| Sub | 1.3677469455214373 |
| 2L/s | 1.0636260670561237 |
| 8L/s | 2.5391998542137797 |
### Chart
| Category | |
|---|---|
| Ctrl | 1.0 |
| Sub | 0.35852469051711533 |
| 2L/s | 0.3103153243712705 |
| 8L/s | 0.4108946167326401 |Relative expression
Relative expression
Ctrl Sub 2L/s 8L/s
Ctrl Sub 2L/s 8L/s
Flooding with physical flow
Flooding with physical flow
Figure S6 Expression of transcripts involved in ROS production, SA synthesis and auxin signaling in plants exposed to submergence and flooding with different flow rate. Ctrl: Control, Sub: Submergence

## Slide 7
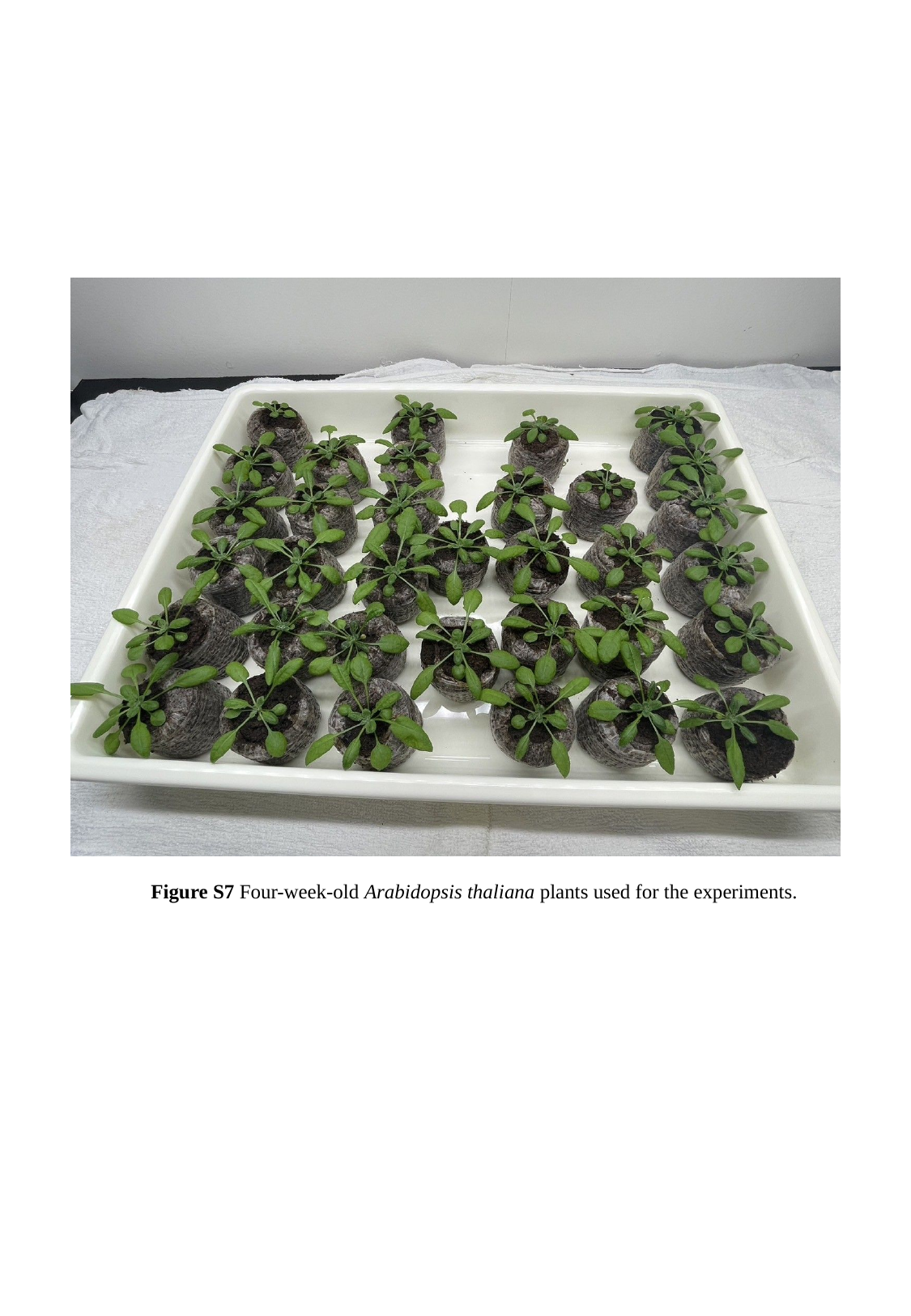

Figure S7 Four-week-old Arabidopsis thaliana plants used for the experiments.
